# Nurse leadership: Sustaining a culture of safety

**DOI:** 10.4102/hsag.v27i0.2009

**Published:** 2022-10-25

**Authors:** Helena E.M. Haskins, Lizeth Roets

**Affiliations:** 1 Department of Health Studies, University of South Africa, Pretoria, South Africa

**Keywords:** nursing leadership, culture of safety, patient safety, cultural diversity, positive work environment

## Abstract

**Background:**

Nurse leaders are essential to manage nursing practices that affect patient safety; therefore, they must create and sustain a sound safety culture in a diverse cultural environment.

**Aim:**

To describe the specific actions required by nurse leadership to enhance the sustainability of a safety culture in hospitals and among a diverse nursing team, ultimately improving patient outcomes.

**Setting:**

Two hospitals in the United Arab Emirates (UAE) were selected purposively, based on the diversity of the nursing team.

**Methods:**

A quantitative design, using Reason’s safety culture framework and Ekenedo’s behavioural safety model, formed the theoretical background of this study to identify the safety culture and positive work environment that exist among culturally diverse nurses. Thirty-four nurse managers and 417 nurses were conveniently selected to participate. Various instruments were used to gather hospital outcomes and other data from respondents pertaining to their demographics, patient safety, positive work environments and safety culture.

**Results:**

Findings received from the nursing team describe the correlation between patient safety, a diverse nursing workforce and positive work environment affecting a safety culture and promoting positive patient outcomes.

**Conclusion:**

Nurse leaders’ integration of specific actions to address the system, as well as diverse nursing teams’ behavioural practices, create a patient care environment that adequately contributes to safety culture practices and enhances positive patient outcomes, which are essential for a culture of safety.

**Contribution:**

The study contributes by providing a structured integration of specific actions for nurse leaders to sustain practices ensuring positive patient outcomes.

## Background

Nurse leaders are essential to manage nursing practices that affects patient safety; therefore, they must create and sustain a sound safety culture in a diverse cultural environment such as the United Arab Emirates (UAE).

With healthcare evolving, there is growing attention and focus on patient safety, ultimately requiring nurse leadership practices that follow through on patient safety events and manage nursing actions that affect patient safety.

A diverse healthcare workforce further complicates matters, and nurse leaders (also referred to as nurse managers) face increased challenges in sustaining patient safety (Padgett et al. 2017).

The behavioural safety culture model of Ekenedo defines the specific skills and decisions that nurse leaders must apply in their departments to sustain a sound safety culture (Besnard et al. 2018; Golda 2013).

A nurse leader has many responsibilities, such as instilling a culture of safety; assessing, reducing, mitigating and managing safety risks in a caring environment; maintaining a safe patient allocation based on the acuity and skill mix of the nurses; evaluating team performances; sharing and educating patient safety measures; and managing risky behaviours among nursing staff (Bronkhorst, Tummers & Steijn 2018; Edgar 2017).

The hospital climate (or ‘positive work environment’, used interchangeably) can be described as the ‘perceived and recurring patterns of behaviour, attitudes and feelings that characterise life in the organization’ (Gea-Caballero et al. 2021). Because nurses spend most of their time in the work environment, facilitating a safe, positive work environment is essential to ensure the safety of patients and staff.

Evidence shows that nurse leaders have a direct impact on the perceived climate based on their commitment to a culture of safety, communication, fostering teamwork, productivity, scheduling and recognition of nurses’ achievements that support patient safety (Farokhzadian, Nayeri & Borhani 2018). It is essential for nurse leaders to understand how their nursing team perceives the hospital climate to proactively address patient safety events in order to promote positive patient outcomes

There are numerous positive work environment tools that the nurse leader can use to determine how their nursing team perceives their work environment, and in the UAE many healthcare organisations apply the National Database of Nursing Quality Indicators nurse surveys annually (Alsalam et al. 2018). Ultimately, this information may provide the nurse leader with supporting information to establish how these factors affect the culture of safety in the unit.

A culture of safety was established as the result of a number of events that occurred in the aviation and nuclear industries (Al-Lawati et al. 2018). However, a culture of safety specifically related to healthcare can be defined in terms of measuring the different dimensions appropriate to clinical settings (Hogden et al. 2017).

Creating an institutional safety culture is not based on a new set of rules but rather on a philosophy that should be embraced by the healthcare team to prevent harm in the workplace (Hogden et al. 2017). Healthcare organisations with a positive safety culture are characterised by communications founded on mutual trust, shared perceptions of the importance of safety and confidence in the efficacy of preventive measures (Hodgen et al. 2017).

Jilcha and Kitaw (2016) and Ma and Rankin ([sa]) list five components required for a sound culture of safety to be applied in healthcare, namely (1) an informed culture, (2) a flexible culture, (3) a reporting culture, (4) a learning culture and (5) a just culture. Applying them in practice allows the nurse leader to address all components in a clinical care setting.

According to Reason’s Culture of Safety Framework (Jilcha & Kitaw 2016; Ma & Rankin [sa]), a learning culture is vital to enable nurses to practise safely; thus, nurses’ orientation, regular in-service training programmes and unit-specific competency training should be offered. In creating an environment of learning, nurses are empowered to assess their own learning needs and practise safely (Oliveira et al. 2017).

As technology advances, so does the complexity of systems within healthcare; therefore, various subjective safety indicators are required to determine the nursing teams’ safety performance (Mirrah et al. 2020).

Currently, there is no benchmarking among the UAE hospitals; thus, quality and services are measured against international hospitals, which do not have the same cultural backgrounds and issues. Healthcare facilities should ultimately benchmark against other facilities in the same country in order to constantly work towards enhanced practices and processes for improved patient safety and patient outcomes.

Patient safety is the reduction of risks associated with injury or harm to a patient to a minimal acceptable level (World Health Organization 2018). Pelzang and Hutchinson (2018) claim many patients are affected by adverse events such as poor documentation practices, falls, insufficient hand hygiene (HH) and hospital-acquired pressure injury (HAPI). Some experience lasting damage, while others could potentially die.

Safety culture practices are not always sustained in hospitals (Alsalam, Bowie & Morrison 2018), despite nurses being exposed to various development, planning and education projects on standards of care and safety practices. Although hospitals use nursing-sensitive performance indicators, the variances reported have had a negative impact on patient outcomes (Alsalam et al. 2018).

On average, in the two hospitals investigated, patient fall rates have increased from 0.1% to 0.4%, HAPI incidences increased from six to eight cases per month, HH compliance was at 85% and the Nursing Admission Assessment (NAA) completed within the specified 24 h was at 90%.

Nurse leaders must understand the synergy between the factors in the hospital climate and safety culture on patient safety to identify the impact it has on positive patient outcomes (Churruca et al. 2021; Willmott & Mould 2018).

Therefore, describing the specific actions required by nurse leaders to facilitate a safety culture and improve patient outcomes may be advantageous to address cultural diversity among the nursing team, as well as promoting a positive work environment and managing safety factors affecting the safety culture.

The purpose of this study is to describe specific actions required by nurse leaders to enhance the sustainability of a safety culture in hospitals and among a diverse nursing team, ultimately improving patient outcomes.

## Methods

Reason’s safety culture framework and Ekenedo’s behavioural safety model (Golda 2013; Jilcha & Kitaw 2016; Ma & Rankin [sa]) formed the theoretical background of this study. Utilising these two theoretical frameworks provided insight into how the study hospitals compared to Reason’s safety culture and the Ekenedo behavioural safety model to clarify reasons for the nursing team not sustaining safe practices.

### Research design

A quantitative design was utilised (Creamer 2018) to identify and describe the hospitals’ safety culture and describe the specific actions that may facilitate a sustainable safety culture among the nursing team that contributes to improved patient outcomes.

### Population and sample

Six hospitals in Abu Dhabi fall under the umbrella of the Department of Health, of which two were purposively selected to gather data, because these hospitals are known to have a diverse nursing workforce. Forty-six nurse managers and 1597 nurses work in these hospitals, of which 34 nurse managers and 417 nurses were conveniently selected to participate. Based on a 95% confidence interval (CI), only 310 nurse respondents were required for a satisfactory sample size.

### Data collection tools

Checklists were used to gather hospital outcome data, and different questionnaires were used to collect data from nurses and nurse managers pertaining to demographics, patient safety, a positive work environment and safety culture.

### Reliability and validity

#### Reliability

Reliability was achieved by adapting an already validated checklist developed by the Abu Dhabi Health Services Company (SEHA) to obtain hospital outcomes data. The self-developed questionnaire, based on a thorough literature review, was pretested after being assessed by a scientific committee of experts and a statistician. The response rate for the nursing respondents’ questionnaires during the pretest was 100%, which is exceptional considering an acceptable response of 80% is described in the literature (Creamer 2018). The descriptive results from Statistical Package for Social Sciences (SPSS; IBM Corporation, Armonk, New York, United States) on all ‘culture of safety questions’ demonstrated a mean of above 3, the standard deviation for all questions ranged between 0.59 and 1.0 and Cronbach’s alpha was 0.877. These results are indicative of the high reliability of the data.

#### Validity

Criterion-related validity was ensured as the researcher sought to establish a relationship between the scores on an instrument and some external construct by comparing the hospital outcome data with the data obtained from nurses and nurse leaders regarding patient safety questions.

Face validity was applicable as the different questionnaires for the nurses and nurse leaders were pretested to determine nurses’ and nurse leaders’ culture of safety and positive work environment practices, and the influence of cultural diversity on the safety culture was compared with the hospital data.

### Data collection methods

The chief nursing officers in each of the study hospitals were recruited to provide patient outcome data recorded on the checklist pertaining to the following aspects: completion of the NAA within 24 h, fall rates, HAPI incidence rates and HH compliance rates.

Research coordinators were appointed in both study hospitals to distribute the information leaflets, questionnaires and consent forms to the participants. Various sessions were scheduled to allow participants to attend the sessions based on their convenience. In total, 900 nurses (600 from hospital A and 300 from hospital B) and 34 (*n* = 46) nurse leaders attended the various information sessions, A 4-week period was provided to return questionnaires in the sealed boxes available on each unit. These boxes were then sealed and collected for data analysis.

### Ethical considerations

Ethical clearance to conduct this study was obtained from the Health Studies Higher Degrees Committee of the University of South Africa (reference number: REC – 012714-039) and the two hospitals’ institutional ethics review boards (reference numbers: REC – 26.02.2015 [RS-357]; REC – AAH/EC-03-15-002). Individual respondents provided written informed consent. The ethical principles of beneficence, nonmaleficence, justice, human dignity, privacy and confidentiality of information, as described by Creamer (2018), were adhered to.

## Results

Statistical Package for Social Sciences 2010 was used to analyse the data received from 417 (46.3% response rate) nurses and 34 nurse leaders (73.91% response rate).

### Biographical data of nurses and nurse leaders

Attum et al. (2020) state that privacy and gender are very important in Islam, and gender was thus an important aspect in this context, where the majority of the nursing team were female (*n* = 345, *f* = 81.03%; *n* = 26, *f* = 76.45%).

As the majority (*n* = 257; *f* = 61.33% nurses and *n* = 33; *f* = 97% nurse leaders) of respondents fell in the 35–64 age group, it was indicative of a sound competence and skills mix (Table 1). The literature describes older nurses as more experienced and skilled because of exposure to clinical settings (Bridges et al. 2019).

**TABLE 1:** Gender and age of nurses and nurse leaders.

Nurses (*n* = 416)	Age	*n*	*f* = %	Nurse leaders (*n* = 34)	Age	*n*	*f* = %
Female *n* = 345	18–24	8	1.92	Female *n* = 26	18–24	0	0.00
	25–34	113	27.20		25–34	1	2.94
	35–44	139	33.41		35–44	8	23.52
	45–54	64	15.38		45–54	12	35.29
	55–64	21	5.04		55–64	5	14.70
Male *n* = 67	18–24	1	0.24	Male *n* = 8	18–24	0	0.00
	25–34	32	7.70		25–34	0	0.00
	35–44	17	4.08		35–44	3	8.82
	45–54	15	3.60		45–54	4	11.76
	55–64	1	0.24		55–64	1	2.94

*Source:* Haskins, H.E.M., 2019, *An action plan to sustaining a culture of safety for positive patient outcomes,* doctoral thesis, University of South Africa, Pretoria, viewed n.d., from http://hdl.handle.net/10500/26185

The data in Table 2 indicates the nurse respondents were from diverse cultural backgrounds; the majority (*n* = 164) were from the Philippines, followed by India (*n* = 155). However, nurse leaders were predominantly from Jordan (*n* = 7; *f* = 21.21 %), South Africa (*n* = 7; *f* = 21.21 %), the Philippines (*n* = 5; *f* = 21.21%) and India (*n* = 5; *f* = 21.21%). The least represented were nurse leaders from Egypt, Finland, New Zealand and Romania (*n* = 1; *f* = 3.03%). Patient admissions data revealed that 75% of patients treated in both study hospitals A and B to be Emirati and 25% non-Emirati (from 19 different nationalities encountered in hospitals). As a result of this diversity, language barriers may have existed because of the majority of nurses and nurse leaders not being able to converse in the Arabic language (*n* = 338 nurses and *n* = 22 nurse leaders). Only 70 nurses and 10 nurse leaders spoke an Arabic dialect – Emirati, Jordanian, Syrian, Sudanese and Palestinian. This illustrates that important patient information may not be retrieved and shared because of language deficits, and because competent translators may not be readily available (Basu, Costa & Jain 2017).

**TABLE 2:** Nationalities of nurses and nurse leaders.

Nationality	Nurses (*n* = 416)		Nurse leaders (*n* = 34)	
	*n*	*f* = %	*n*	*f* = %
Jordanian	21	5.05	7	21.21
Filipino	164	39.42	5	15.15
Indian	155	37.25	5	15.15
South African	9	2.16	7	21.21
Emirati	6	1.44	2	6.06
Malaysian	1	0.20	0	0.00
British	4	0.96	0	0.00
German	1	0.20	0	0.00
Pakistani	2	0.24	0	0.00
Syrian	1	0.20	0	0.00
Egyptian	3	0.72	1	3.03
Palestinian	14	3.36	0	0.00
Lebanese	2	0.48	0	0.00
Somali	12	3.36	0	0.00
Sudanese	6	1.44	0	0.00
Bangladeshi	3	0.72	0	0.00
Omani	5	1.20	0	0.00
Seychellois	1	0.20	0	0.00
Australian	1	0.20	0	0.00
Indonesian	3	0.72	0	0.00
Bulgarian	1	0.20	0	0.00
British	0	0.00	3	9.09
Finish	0	0.00	1	3.03
New Zealander	0	0.00	1	3.03
Romanian	0	0.00	1	3.03

*Source*: Haskins, H.E.M., 2019, *An action plan to sustaining a culture of safety for positive patient outcomes*, doctoral thesis, University of South Africa, Pretoria, viewed n.d., from http://hdl.handle.net/10500/26185

Communicating across cultural boundaries increases the risk of misunderstanding, and it is further compounded when dealing with complex scientific and medical information (Brooks et al. 2019). Patient information is in English, but only 5.8% of the nurses’ home language is English, which could have an impact on effective communication and how the nurses apply safety best practices. This finding reflects that nurse leaders should facilitate strategies to assist nurses to improve their English proficiency, to use specific tools to communicate with patients with limited English proficiency and to ensure translators are available in the units (Basu et al. 2017; Brooks et al. 2019).

### Cultural perceptions of nurses and nurse leaders

Nurses (*n* = 339; *f* = 81.5%) and nurse leaders (*n* = 28; *f* = 82.5%) indicated that the cultures of the nursing team and patients directly affect patient safety. The reasons mentioned by the nursing team as to why their own and patient cultures affect patient safety included differences in education, language barriers and cultural values among the nurse and the patient.

The UAE has more than 200 different nationalities residing in the country; thus, healthcare professionals should become aware of the cultural influences and health behaviours related to illness and recovery and translate that awareness into culturally congruent care (Brooks et al. 2019).

The UAE cultural structure consists of each family, traditionally bound by obligations of mutual assistance to their immediate relatives and the tribe as a whole. The culture and traditions of the UAE are grounded in the Islamic heritage of the Arab region, and they seek the Qur’an as a healing source in times of psychological and spiritual distress (UAE webpage).

Cultural aspects can impede patient care plans; therefore, healthcare professionals must understand what drives the patient and family towards healing. Although the Crescent of Care model was developed to guide the care of Arab Muslim patients and describes the holistic care nurses should practise in a daily plan of care, it is not evident in practice in the UAE (Lovering 2012).

Privacy and gender separation are important aspects of Islam, and nurse leaders need to ensure the environment is appropriate and include nurses of the correct gender according to patients’ genders (Attum et al. 2020; Hassan et al. 2020). Appropriate signs need to be displayed, indicating the allowed gender to use a specific room or enter that room to provide care. Knocking on the door before entering to ensure female Muslim patients are appropriately covered is another important cultural principle (Attum et al. 2020).

To ensure that nurses are able to apply transcultural nursing care in practice, senior nurse leadership must confirm that the orientation programmes and ongoing education address cultural differences and expectations (Kaihlanen, Hietapakka & Heponiemi 2019; Uman, Edfors & Jakobsson 2020).

### Culture of safety and nurse leadership

Organisations with a positive safety culture are characterised by communications founded on mutual trust, shared perceptions of the importance of safety and confidence in the efficacy of preventive measures (Hodgen et al. 2017). Jilcha and Kitaw (2016) and Ma and Rankin [sa] list five components required for a culture of safety, namely (1) an informed culture, (2) a flexible culture, (3) a reporting culture, (4) a learning culture and (5) a just culture.

Authors indicate a definite association between a safety culture, a positive work environment and patient safety outcomes in healthcare facilities (Khoshakhlagh et al. 2019; Kumbi et al. 2018).

A five-point Likert scale was used for nurses to grade their perception of the impact a positive work environment, cultural aspects and patient safety have on the sustainment of a culture of safety in the clinical setting.

Nurse leaders must establish an environment of transparency and engage the nursing team in patient safety and safety improvement activities, regularly share patient performance outcome data, ensure open communication, enforce teamwork and provide a supportive environment to speak freely about safety concerns on units, to ensure mutual respect and recognition of nurses’ safety efforts (Khoshakhlagh et al. 2019; Kumbi et al. 2018).

**FIGURE 1:**
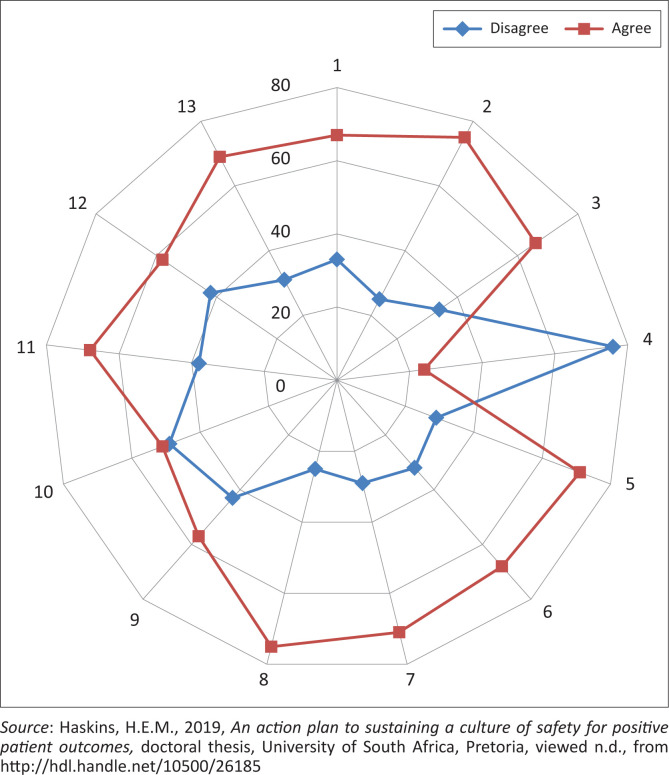
Nurses’ perception of a positive work environment affecting patient safety.

The web diagram in Figure 1 shows nurses did not perceive their work environment as positively supporting patient safety, which is a significant safety concern for nurse leaders to consider (Murray, Sundin & Cope 2017). Moreover, safety culture forms part of larger organisational processes, and nurse leaders must review these in terms of the nurses’ perceptions of the hospital’s environmental safety features that may influence their overall safety performance (Murray et al. 2017).

**FIGURE 2:**
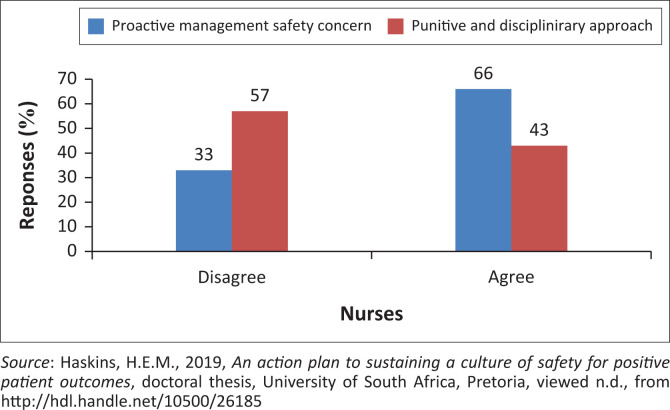
Relation between nurses’ perception of nurse leaders’ proactive system review versus punitive approach.

As illustrated in Figure 2, approximately 57% of nurses confirmed that a punitive environment does not exist within the facility. However, 43% of nurses reported nurse leaders follow a punitive approach if an incident occurred, and 33% disagreed that nurse leaders proactively address system concerns affecting patient safety. This is of concern because a culture of safety is unsustainable if nurse leaders do not address systems and processes.

Based on the nurses’ perception, nurse leaders have a primary responsibility to identify process changes to sustain a sound safety culture in the clinical areas. Nurse leaders must proactively identify system gaps and ensure a safe care environment that could influence patient safety; thus, they need to be visible in the units and have sound decision-making concern for patient safety events (Golda 2013).

As illustrated in Table 3, the majority of nurse respondents agreed (*n* = 279) that nurse leaders are visible and supportive in the units to enable them to address patient safety concerns. However, 134 nurses disagreed (*n* = 64; *f* = 15.4%) and remained neutral (*n* = 70; *f* = 16.8%), which indicates nurse leaders are not always visible and supportive in addressing a safe care environment; such behaviour could have a potential negative impact on patient safety.

**TABLE 3:** Nurse leader’s visibility and support to ensure a safe care environment.

Scale	*n*	*f* = %
Strongly disagree	5	1.2
Disagree	59	14.2
Neutral	70	16.8
Agree	230	55.3
Strongly agree	49	11.8

**Total**	**413**	**99.3**

*Source:* Haskins, H.E.M., 2019, *An action plan to sustaining a culture of safety for positive patient outcomes*, doctoral thesis, University of South Africa, Pretoria, viewed n.d., from http://hdl.handle.net/10500/26185

It is essential for nurse leaders to apply ‘just culture’ principles in practice to eliminate patient safety concerns, address system issues and manage unsafe practices among the nurses (Haskins 2019; Paradiso & Sweeney 2019). Nurse leaders must (1) follow clear disciplinary processes, (2) frequently review system issues, (3) manage system defects through frequent processes review, (4) use structured performance reviews to manage nurses’ behaviour affecting patient safety, (5) obtain data from the incident reporting system allowing them to manage patient safety issues, (6) support hospital management in addressing system issues and (7) manage incidents through appropriate quality methodologies.

**FIGURE 3:**
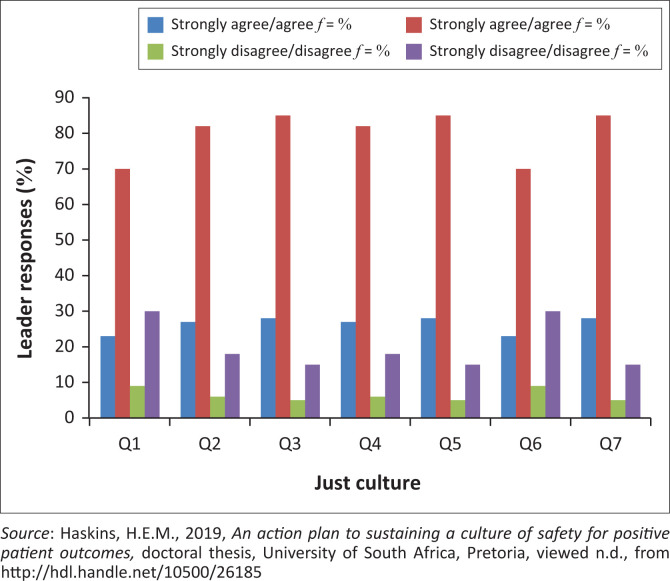
Nurse leaders’ just culture practices.

It is evident in Figure 3 that most nurse leaders (*n* = 26 of *n* = 33) applied the basics of ‘just culture’ in managing nurses’ behavioural practice gaps. However, a third of the respondents disagreed, indicating that nurse leaders did not utilise ‘just culture’ practices within the context of patient care.

## Discussion

Nurse leaders have a significant role and responsibility for ensuring the sustainment of a culture of safety despite a diverse nursing workforce responsible for nursing care. An analysis of the data within this research study revealed that nurse leaders must take specific actions to sustain a culture of safety, thereby improving patient outcomes.

English proficiency testing of nurses prior to employment must be assessed, and remedial opportunities to provide basic Arabic or any other relevant language proficiency training when joining a hospital or any other healthcare facility where diversity exists must available. This will allow for communicating among a diverse nursing team (Al-Harasis 2012; Oducado et al. 2020).

Specific tools to communicate with patients with limited English proficiency or availing competent translators to assist with the communication are required. Translators might be a feasible solution to the challenges experienced; however, availability and cost implications might be challenging in resource-poor healthcare environments (Blay et al. 2018).

Nurse leaders must ensure that induction programmes include cultural training to enable the diverse team to provide culturally congruent care. Induction programmes and continuous professional development opportunities focusing on basic healthcare education and communication in more than one language will be useful to allow patients and professionals to understand one another, contributing to a safety culture (Noonea et al. 2020; NSW Report [sa]).

It is important that nurse leaders should create and facilitate a positive work environment (hospital climate) that promotes leadership support and visibility, reward and recognition, effective communication, teamwork, safe staffing and patient allocation for staff to ensure patient safety. This will facilitate a work environment where nurses feel free to engage, speak up and participate in safety events that might affect patient safety (Debika, Kumar & Kumari 2020).

Nurse leaders must practise a culture of safety through sharing information related to patient safety. They must be flexible in managing patient safety and report incidents or concerns that could have an impact on patient safety. They have to apply a ‘just culture’ to manage behavioural choices and system safety as well as providing opportunities for a continuous learning culture among their diverse nursing team. This will lead to nursing team being proactive, engaged, empowered and educated on safety event prevention that will facilitate sustainability of a safety culture (Churruca et al. 2021).

Nurse leaders must furthermore create a ‘just’ culture by following a clear disciplinary process. They must continuously review system concerns affecting safety, manage system defects through frequent process reviews, use structured performance reviews to manage nurses’ behaviour affecting patient safety and obtain data from the incident reporting system to manage patient safety issues. They also have to support hospital management in addressing system issues and manage incidents through appropriate quality methodologies. It is therefore essential for nurse leaders to practise ‘just’ culture to allow the nursing team to feel engaged, educated and empowered to manage their own practice and speak up if safety concerns are present in the care environment and thus model optimal clinical outcomes (Fend, Willoughby & Jackson 2021; Paradiso & Sweeney 2019).

The strengths of the study describe the importance that nurse leaders have to play to sustain a culture of safety, thereby ensuring positive patient outcomes. It is of value to do further research to determine the direct correlation that the diverse culture might have on sustaining a culture of safety.

This study described the structured integration of specific actions for nurse leaders.

## Conclusions

Nurse leaders must understand the influence that the hospital climate factors, cultural diversity, patient safety risk and ‘just’ culture practices have on positive patient outcomes and sustaining a culture of safety.

It is therefore important for nurse leaders to ensure that patient care environments adequately contribute to safety culture practices that enhance positive patient outcomes through the application of specific actions in operational activities. It is possible to sustain a culture of safety if very specific actions are implemented and facilitated in practice to allow patients and professionals from diverse cultures to enhance a culture of safety, ultimately improving patient outcomes and providing a positive patient experience.
